# Backbone NMR assignment of the nucleotide binding domain of the *Bacillus subtilis* ABC multidrug transporter BmrA in the post-hydrolysis state

**DOI:** 10.1007/s12104-021-10063-2

**Published:** 2022-01-05

**Authors:** Victor Hugo Pérez Carrillo, Dania Rose-Sperling, Mai Anh Tran, Christoph Wiedemann, Ute A. Hellmich

**Affiliations:** 1 Faculty of Chemistry and Earth Sciences, Institute of Organic Chemistry and Macromolecular Chemistry, Friedrich Schiller University Jena, Humboldtstraße 10, 07743 Jena, Germany; 2 Centre for Biomolecular Magnetic Resonance (BMRZ), Goethe University, Max von Laue Str. 9, 60438 Frankfurt, Germany; 3 Cluster of Excellence “Balance of the Microverse”, Friedrich Schiller University Jena, 07743 Jena, Germany

**Keywords:** Multidrug resistance, Microbial transporter, Catalytic cycle, ADP-bound state, Post-hydrolysis, Solution NMR

## Abstract

ATP binding cassette (ABC) proteins are present in all phyla of life and form one of the largest protein families. The *Bacillus subtilis* ABC transporter BmrA is a functional homodimer that can extrude many different harmful compounds out of the cell. Each BmrA monomer is composed of a transmembrane domain (TMD) and a nucleotide binding domain (NBD). While the TMDs of ABC transporters are sequentially diverse, the highly conserved NBDs harbor distinctive conserved motifs that enable nucleotide binding and hydrolysis, interdomain communication and that mark a protein as a member of the ABC superfamily. In the catalytic cycle of an ABC transporter, the NBDs function as the molecular motor that fuels substrate translocation across the membrane via the TMDs and are thus pivotal for the entire transport process. For a better understanding of the structural and dynamic consequences of nucleotide interactions within the NBD at atomic resolution, we determined the ^1^H, ^13^C and ^15^N backbone chemical shift assignments of the 259 amino acid wildtype BmrA-NBD in its post-hydrolytic, ADP-bound state.

## Biological context

In bacteria, the enhanced expression and production of efflux pumps can lead to multidrug resistance (MDR) which is a (re)emerging problem for global human health. ATP binding cassette (ABC) transporters are an important family of MDR conferring membrane proteins and the only primary active transporters implicated in this phenomenon (Henderson et al. 2021).

In 2004, the *Bacillus subtilis yvcC* gene was found to encode a constitutively expressed ABC transporter (Steinfels et al. 2004). Due to its ability to extrude the fluorescent dyes Hoechst 33342, doxorubicin, and 7-aminoactinomycin D and its similarity to the human and lactococcal multidrug ABC transporters P-glycoprotein and LmrA, it was aptly renamed BmrA (Bacillus multidrug resistance ATP). BmrA has since become a poster child to study drug efflux and the molecular mechanisms of (bacterial) ABC exporters by numerous biophysical techniques (e.g. Dalmas et al. 2005a; Do Cao et al. 2009; Mehmood et al. 2012; Kunert et al. 2014; Wiegand et al. 2017; Lacabanne et al. 2019).

The functional BmrA protein is a 129 kDa homodimer (Ravaud et al. 2006). Each monomer contains a transmembrane domain (TMD) and a nucleotide binding domain (NBD) of 28.5 kDa. The TMDs are responsible for substrate recognition and translocation while the NBDs mediate ATP binding and hydrolysis which triggers the conformational changes of the transporter’s conformational cycle. In the simplest terms, this cycle begins when ATP binding induces NBD dimerization, which leads to a switch from an inward to an outward facing conformation in the TMDs. Upon nucleotide hydrolysis, the transporter is “reset” and the cycle can begin anew (Szöllősi et al. 2018).

Whereas the amino acid sequence and structure of the TMDs varies among members of the ABC superfamily, the NBDs are highly conserved. Nonetheless, the molecular details of how ABC transporter NBDs sense nucleotides and allosterically mediate conformational changes remain under debate (Szöllősi et al. 2018). The NBD structurally contains two subdomains, the RecA-like subdomain which includes the Walker A and B motifs and the Q-, D-, and H-loops, and the α-helical subdomain containing the signature motif (ABC motif/C-loop) and the X-loop (Orelle et al. 2019). Similar to other ABC transporters, both the BmrA-NBD Q-loop and the X-loop, directly N-terminal to the C-loop, have been implicated in interdomain communication (Dalmas et al. 2005b; Lacabanne et al. 2019). Upstream of the Walker A motif, an aromatic residue termed the A-loop is responsible for interacting with the nucleotide base (Ambudkar et al. 2006). Together, all of these conserved motifs are responsible for ATP binding and hydrolysis as well as NBD-NBD and NBD-TMD communication and even seem to be responsible for the unidirectionality of substrate transport in ABC transporters (Grossmann et al. 2014; Xu et al. 2017).

Following low-resolution cryo-electron microscopy (cryo-EM) work on BmrA (Chami et al. 2002; Orelle et al. 2008; Fribourg et al. 2014), recently X-ray and cryo-EM structures for the full-length protein in the outward facing conformation became available at 3.95 Å (pdb: 6R72) and 3.90 Å resolution (pdb: 6R81), respectively (Chaptal et al. 2021). As is frequently necessary in structural studies of ABC transporters (Ford und Hellmich 2020), an ATPase inactivating mutation in the NBD was required for the stabilization of the protein to obtain these structures in the MgATP-bound state (Chaptal et al. 2021). In the case of BmrA and related bacterial ABC transporters, spectroscopic approaches including solid-state NMR and EPR thus provide additional valuable insights into the global conformational dynamics of the (wildtype) systems, the role of lipids and the interactions with substrates and nucleotides (Hellmich et al. 2008, 2012b; Hellmich und Glaubitz 2009; Zou et al. 2009; Kunert et al. 2014; Kaur et al. 2016, 2018; Neumann et al. 2017; Wiegand et al. 2017; Spadaccini et al. 2018; Rose-Sperling et al. 2019). Nonetheless, the molecular details of the local dynamic and structural consequences of nucleotide binding to the NBD as the central step of the ABC transporter catalytic cycle typically remain unresolved, either due to the use of site-specific labels or the significant signal overlap that such a large system evokes which typically precludes obtaining per residue information within the NBD upon nucleotide binding or hydrolysis. Such issues can be circumvented with complementary solution NMR studies on the isolated NBDs. Importantly, the NBDs of ABC transporters are stable in isolation and maintain their structural and functional integrity, i.e. their ability to interact with nucleotides. In the case of BmrA and its close relative LmrA, the NBDs are monomeric in the absence of the TMD thus making them especially amenable to solution NMR studies (Hellmich et al. 2015). Overall, the NBDs of ABC transporters present valuable systems to investigate the molecular consequences of nucleotide interaction without the interference of crosstalk with other domains (Ford und Hellmich 2020). In line with our previous studies on the LmrA-NBD (Hellmich et al. 2012a), we noticed that due to intrinsic protein dynamics, several resonances are missing from the ^1^H, ^15^N-HSQC spectrum of ^15^N labeled BmrA-NBD in the apo state, i.e., without nucleotide. However, these resonances appear upon addition of ADP. Here, we report the backbone resonance assignments of the isolated wildtype (WT) BmrA-NBD (residues G331-G589) in its post-hydrolysis, ADP-bound state as a model system for the detailed structural and dynamic analysis of the ABC transporter NBD as one of the most prominent functional domains found in all phyla of life.

## Methods and experiments

### Protein expression and purification

A synthetic gene coding for WT BmrA-NBD (residues G331-G589) with a TEV cleavage site following a (His)_6_-tag was obtained from GenScript (Piscataway Township, NJ, USA) and cloned into pET11a vector. Transformed *E. coli* BL21 gold (DE3) cells were grown at 37 °C until an OD_600_ of 0.6 was reached, induced with 1 mM IPTG and grown overnight at 21 °C. Triple ^2^H, ^13^C and ^15^N isotope labeled protein was obtained by growing cells in commercially available Silantes OD2 *E. coli* medium (Silantes GmbH, Munich, Germany). For amino acid specific labeling, defined medium (Muchmore et al. 1989) was complemented with either ^15^N-labeled lysine, arginine, tryptophane, tyrosine, phenylalanine, valine, leucine, isoleucine or serine and glycine (Cambridge Isotope Labs, Tewksbury, MA, USA) in addition to the remaining 19(18) amino acids in their unlabeled form. Cells were harvested by centrifugation (5000×*g*, 10 min, 4 °C). The cell pellet was frozen in liquid nitrogen and stored at − 20 °C until further use.

For purification, the cell pellet was dissolved in lysis buffer (250 mM Sucrose, 150 mM NaCl, 2.5 mM MgSO_4_, 10 mM Tris/HCl pH 7.5 and 1 mM DTT) supplemented with a protease inhibitor cocktail, DNase, RNase, lysozyme and benzamidine (all from Sigma Aldrich). Cells were disrupted with a cell homogenizer (Bandelin, Berlin, Germany). Membranes and cell debris were pelleted at 20,000 rpm, 30 min, 4 °C and the supernatant containing WT BmrA-NBD was loaded onto a NiNTA column (Qiagen, Hilden, Germany) previously equilibrated with 50 mM Tris/HCl pH 8, 50 mM NaCl. After washing with 10 CV of buffer A (50 mM Tris/HCl pH 8), 10 CV of buffer B (50 mM Tris/HCl pH 8, 500 mM NaCl) and 10 CV of buffer C (Tris/HCl pH 8, 50 mM NaCl, 20 mM imidazole pH 8), WT BmrA-NBD was eluted with 5 CV of elution buffer (Tris/HCl pH 8, 50 mM NaCl, 250 mM imidazole pH 8). After addition of His-tagged TEV protease (1:40 mol/mol) to remove the His-tag from WT BmrA-NBD, the proteins were dialyzed overnight at 4 °C in 50 mM BisTris pH 7, 50 mM NaCl. Dialyzed protein was then loaded onto a fresh NiNTA column. The flow through was collected and the column was washed with 6 CV of buffer A to obtain the maximum amount of tag-free WT BmrA-NBD. The protein was concentrated and loaded onto a size exclusion column (HiLoad 16/600 Superdex 200 pg, Cytiva, Freiburg, Germany). The fractions containing WT BmrA-NBD were collected and sample purity was verified by SDS-PAGE.

### NMR spectroscopy

For NMR experiments, samples were concentrated to 250–400 µM before addition of 10 mM ADP and 10% v/v D_2_O and 0.15 mM DSS (final concentrations). NMR spectra of isotope labeled BmrA-NBD in 50 mM BisTris pH 7, 50 mM NaCl were recorded at 298 K on Bruker AVANCE 600, 800, and 900 MHz spectrometers equipped with cryogenic triple resonance probes (Bruker GmbH, Karlsruhe, Germany). TROSY-based ^15^N-HSQC, HNCA, HNCO, HN(CA)CO, HN(CO)CA, HNCACB and HNCOCACB experiments were recorded using standard pulse sequences (Salzmann et al. 1998). All spectra were processed using Bruker TOPSPIN 4.0.8 and analyzed using CARA (Keller 2004).

The secondary structure of WT BmrA-NBD based on the backbone chemical shift assignment for the ADP-bound state was determined with TALOS-N (Shen and Bax 2013).

## Extent of assignment and data deposition

The nucleotide binding domains (NBDs) are highly conserved across the entire ABC protein superfamily. However, the structural and dynamic details of how ATP binding and hydrolysis in the NBDs is communicated to the TMDs and thus enables substrate transport are not yet fully understood. We chose the 259 amino acid NBD of the *B. subtilis* ABC transporter BmrA (residues G331-G589) as a model for an in-depth NMR spectroscopic investigation of these questions. Letting the native residue G331 moonlight as the final residue of an N-terminal TEV-cleavage site, we were able to obtain completely tag-free WT BmrA-NBD without any residual, non-native residues. BmrA-NBD chemical shift assignments were determined using ^2^H, ^13^C, ^15^N-labeled protein supplemented with 10 mM ADP, and verified or augmented using selectively labeled ^15^N-Lys, ^15^N-Phe, ^15^N-Tyr, ^15^N-Arg, ^15^N-Val, ^15^N-Leu, ^15^N-Ile, ^15^N-Trp and ^15^N-Ser/^15^N-Gly labeled WT BmrA-NBD.

**Fig. 1:**
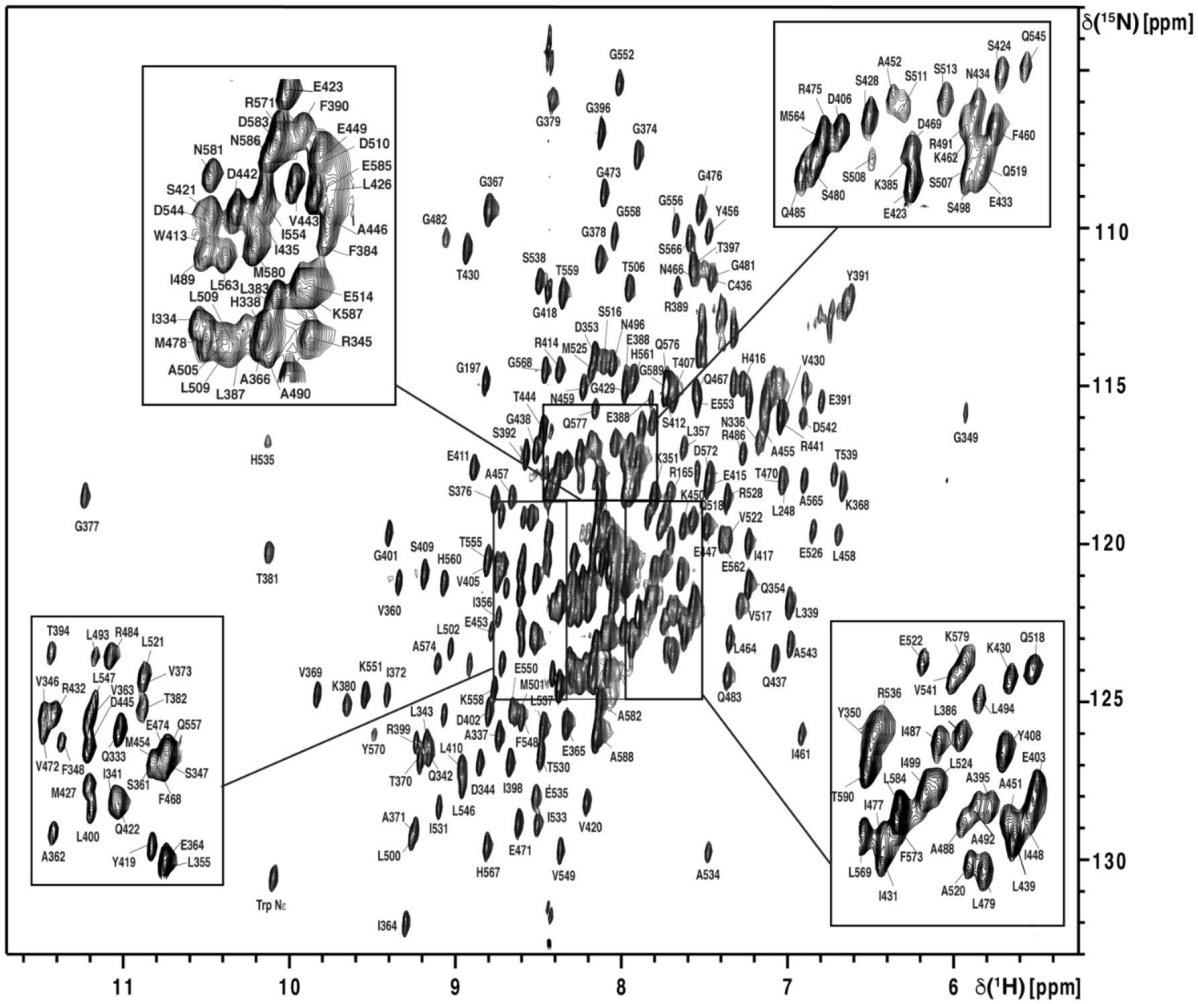
2D-^1^H, ^15^N-TROSY-HSQC spectrum of ^2^H, ^15^N, ^13^C-labeled BmrA-NBD recorded at 298 K on a 600 MHz spectrometer equipped with a cryogenic triple resonance probe (Bruker GmbH, Karlsruhe, Germany), using a 280 µM sample. The assignments are given in single letter code following the numbering scheme for the full-length BmrA ABC transporter. Note the atypical chemical shifts observed for residues belonging to the Walker A motif (G377, T381) and the H-loop (H535) between 9.25/115 and 11.25/122.5 ppm (^1^H and ^15^N chemical shift, respectively)

For 97.2% of all non-proline residues, complete backbone assignments were obtained (Fig. 1). WT BmrA-NBD has eight proline residues. Except for P425, the C′, Cα and Cβ chemical shifts could be determined for all of them. For the remaining amino acids with unassigned and/or missing NH resonances, C′, Cα and Cβ chemical shifts have been determined for K332, L426, E440, E504, Q512, and K515. This leaves only P452 and the proximal N-terminal residue G331 with no chemical shift information at all.

With the exception of the catalytic glutamate residue of the Walker B motif (consensus sequence hhhhDE with h = hydrophobic amino acid), with the sequence ^499^ILMLDE^504^ in BmrA, for which the backbone NH resonance is not available, the assignments of all other conserved motifs within the NBD are complete. This includes the other two important motifs designating a protein as an ABC superfamily member, the Walker A (consensus sequence GxxGxGK(S/T) with x = any amino acid), and C-loop (LSGGQ(K/R)Q), i.e. residues ^374^GPSGGGKT^381^ and ^479^LSGGQRQ^485^ in BmrA, respectively. The H-loop residues ^533^IAHRL^537^ (consensus sequence hAHRL, with h = hydrophobic residue) are also fully assigned. As previously observed in ^1^H, ^15^N-HSQC spectra of the NBD of *L. lactis* LmrA in the ADP-bound state (Hellmich et al 2012a), atypical chemical shifts are observed for resonances from residues belonging to the Walker A motif (G377, T381), and the H-loop (H535). Presumably the close proximity to the phosphate groups of ADP leads to these peak shifts. Finally, the residues constituting the A-loop (consensus sequence (F/K)xY), ^348^FGY^350^, the Q-loop (hV(S/P)Q), ^419^YVSQ^422^, the D-loop (SALD), ^507^SSLD^510^ and the X-loop (TRVGDKGTQ), ^470^TEVGERG^476^, are also fully assigned.

**Fig. 2:**
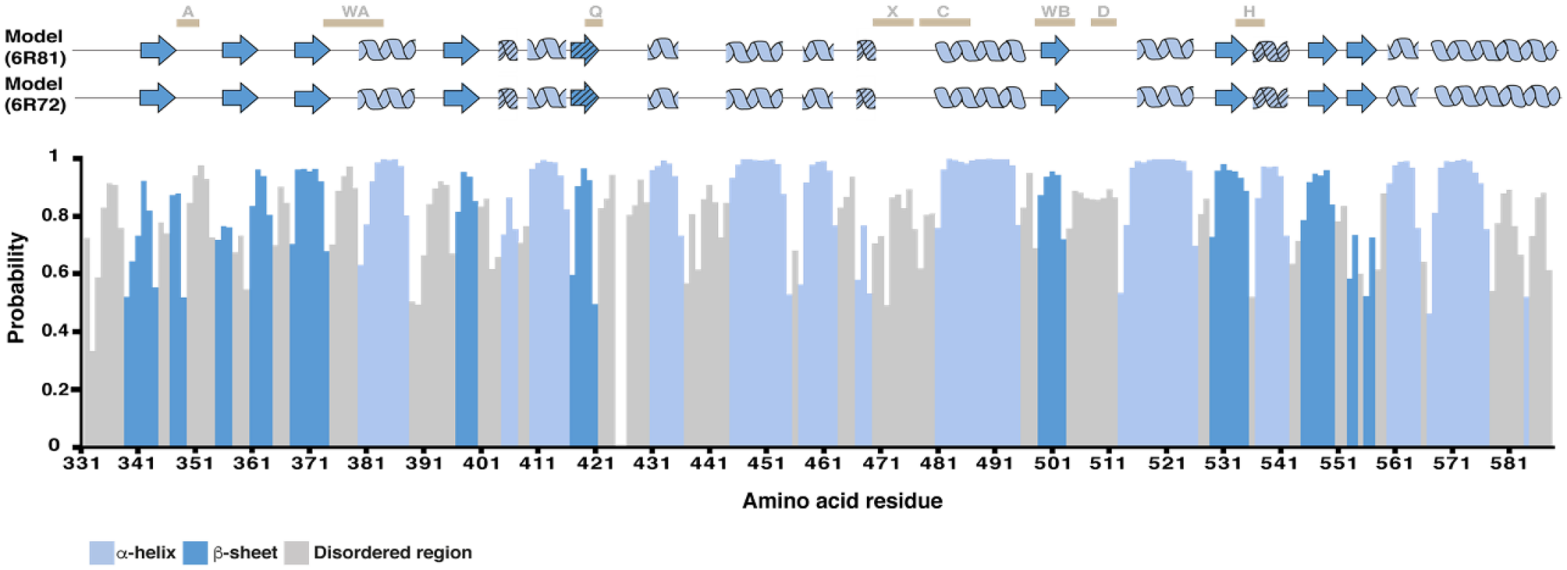
Chemical shift based secondary structure prediction of the WT BmrA-NBD in the ADP-bound state using TALOS N (Shen and Bax 2013) The secondary structure has been compared with the two structures available for BmrA in the outward open state as shown with the topology model on top (PDB: 6R81, 6R729; Chaptal et al. 2021). Residues with lower secondary structure propensity are marked with hatched symbols. Blank spaces in the secondary structure probability plot below represent unassigned amino acids. A: A-loop, WA: Walker A, Q: Q-loop, X: X-loop, C: C-loop, WB: Walker B, D: D-loop, H: H-loop

We used the chemical shift assignments to determine the secondary structure of the BmrA-NBD in solution using TALOS-N (Shen und Bax 2013) (Fig. 2) and compare it to the newly available X-ray and cryo-EM structural data of the NBDs in the context of full-length BmrA (Chaptal et al. 2021). Overall, our secondary structure prediction is a close match to both the cryoEM structure (pdb: 6R81) and the X-ray structure (pdb: 6R72). Notably, the full-length structures made use of an ATP hydrolysis deficient Walker B mutation, E504A, to obtain the outward open conformation with dimerized NBDs. Both full-length structures of dimeric, ATP*Mg^2+ ^-bound BmrA are resolved to the very last C-terminal residue, and the C-terminus is α-helical throughout. Previously, the C-terminus of ABC exporters has been implicated in mediating NBD-NBD interactions (Nöll et al. 2017). In the case of the heterodimeric *T. thermophilus* ABC transporter TmrAB, it was postulated that the C-terminal helices can adopt both a side-by-side as well as a crossing over conformation, both of which are implicated in rearrangements of the NBDs as the transporter moves through its substrate translocation and ATP hydrolysis cycle (Nöll et al. 2017). In the BmrA structures, the two α-helical C-terminal helices also cross over to the adjacent NBD (Chaptal et al. 2021). Here, based on our NMR data of the isolated, monomeric, ADP-bound NBD, the C-terminus becomes disordered (Fig. 2). While it remains to be seen whether this is because we are observing the post-hydrolysis ADP-bound state or due to the absence of the TMD, it indicates that the C-terminal region of ABC transporter NBDs may be even more flexible than previously anticipated and highlights the importance of studying ABC transporter domains both in isolation and in the context of full-length transporters.

In summary, with the near-complete backbone chemical shift assignment of the BmrA-NBD, we provide the basis for an in-depth analysis of the structural and dynamic changes in the NBD of an ABC transporter at atomic resolution and provide first insights into the putative structural consequences of interdomain interactions.

## Data Availability

The assignments of the wildtype BmrA-NBD in the ADP-bound state have been deposited in the BioMagResBank (https://bmrb.io/) under the accession number 51156.
